# Prediction of Early Recurrence of Solitary Hepatocellular Carcinoma after Orthotopic Liver Transplantation

**DOI:** 10.1038/s41598-019-52427-8

**Published:** 2019-11-01

**Authors:** Jiliang Feng, Ruidong Zhu, Dezhao Feng, Lu Yu, Dawei Zhao, Jushan Wu, Chunwang Yuan, Junmei Chen, Yan Zhang, Xiu Zheng

**Affiliations:** 1 Clinical-Pathology Center, Bejing You-An Hospital, Capital Medical University, Beijing, People’s Republic of China; 2 General Surgical Center, Bejing You-An Hospital, Capital Medical University, Beijing, People’s Republic of China; 3 Vantage College, University of British Columbia, Vancouver, Canada; 4 Medical Imaging Department, Bejing You-An Hospital, Capital Medical University, Beijing, People’s Republic of China; 5 Department of Interventional Therapy, Bejing You-An Hospital, Capital Medical University, Beijing, People’s Republic of China; 6 Medical Laboratory Center, Bejing You-An Hospital, Capital Medical University, Beijing, People’s Republic of China

**Keywords:** Predictive markers, Hepatocellular carcinoma, Surgical oncology, Risk factors

## Abstract

Hepatocellular carcinomas(HCC) consisted of heterogeneous subtypes with different recurrence probabilities after liver transplantation(LT). Our study aimed to develop an improved model for predicting the recurrence of solitary HCC after LT. In this retrospective study, 151 solitary HCC patients who received orthotopic LT over a period of 10 consecutive years were included. All recipients received graft from deceased donors. The first eligible 50 patients were used as validation cohort and others were utilized to construct the model. A two-tailed P < 0.05 was considered to indicate statistical significance for all analysis. Based on the maximisation of the Youden’s index, the optimal cutoff values for alpha-fetoprotein(AFP) and tumor diameter were 261.6 ng/mL and 3.6 cm, respectively. Vascular involvement includes gross and microscopic vascular invasion. Variables potentially affecting recurrence-free survival(RFS) were examined using univariate and multivariate Cox regression analysis. Univariate and multivariate analysis revealed that AFP, tumor diameter, vascular invasion and cytokeratin-19/glypican-3 sub-typing were independent prognostic factors for RFS, thus comprised the risk scoring model. The AUC values of the model in the cohorts were significantly higher than that of the Milan, UCSF, Fudan and Hangzhou criteria. These findings suggest the model has high performance in predicting early recurrence of solitary HCC patients after LT.

## Introduction

Liver transplantation (LT) is considered as potential curative option for hepatocellular carcinoma (HCC), but its efficacy depends on the risk of recurrence^1^. Over the past two decades, the optimization of preoperative recipient selection, such as Milan criteria (MC) and BCLC (Barcelona Clinic Liver Cancer) staging system has greatly reduced the recurrence rate^2,3^. Although some investigators have argued that MC and BCLC staging system were too restrictive and limited the transplantation option, it was a fact, that recurrence rate of patients who met MC or BCLC (stage 0 to A3) in the first three years after LT was 8–15% and 11%, respectively^4,5^. Most post-LT recurrences occurred within 2 years because of pre-transplant dissemination of primary tumor cells. However, in the third year after LT, intrahepatic and extrahepatic recurrences still occur, although this represents only a small fraction of the overall primary tumor recurrence. Because of the use of immunosuppressive agents or the persistence of tumorigenic factors after LT, there is a risk of de novo tumors, which are most likely to occur after 3 years of transplantation^6–8^. Therefore, we only analyzed the post-LT recurrence within 3 years in this study.

HCC is mainly spread through blood vessels, especially the portal vein. Therefore, in almost all recipient selection criteria, macrovascular involvement and a certain degree of tumor burden with respect to tumor size and number in preoperative imagines were used as an exclusion indicator for LT^6^. However, extrahepatic spread of primary tumors is not always detectable by preoperative imaging examination, which eventually leads to post-LT recurrence^9^. Still, vascular invasion observed in explanted liver or biopsy is of great value in predicting extrahepatic spread and post-transplant recurrence.

Recently, multi-dimensional integrated approach with tumor number, size and biomarkers, such as alpha-fetal protein (AFP), epithelial cell adhesion molecule (EpCAM), neural cell adhesion molecule (NCAM), delta-like 1 homolog (DLK1), and cytokeratin 19 (CK19), lens cullinaris agglutin-reactive AFP (AFP-L3) and des-carboxyprothrombin (DCP), *et al*. showed a great potential as diagnostic and prognostic tool in HCC^10,11^. Of note, most of the biomarkers are associated with liver development and differentiation of hepatic progenitor cell (HPC).

Confirmation of the hierarchical constitution of the parenchymal cells in the adult liver promotes an understanding of the cellular origin of HCC. It is now considered that HCC is composed of heterogeneous subtypes that may be transformed from HPC, as well as the HPC-derived progenies towards maturation^12,13^. Cytokeratin 19 (CK19) and glypican 3 (GPC3) are common diagnostic markers for liver malignancies. In normal condition, CK19 is expressed in HPCs and early committed hepatocytes. With further differentiation, the expression of CK19 disappears rapidly^14–16^. In addition to cholangiocellular carcinoma, CK19 can be detected in some HCC subtypes, indicating that the CK19-positive HCC can originate from HPC^17,18^. GPC3 was abundantly expressed in HPCs and immature hepatocytes but not in mature hepatocytes^19–21^. In well differentiated HCC, GPC3 expression was weak or absent^22,23^. Therefore, according to the specific expression spectrum of CK19 and GPC3 in the differentiation process of HPC towards mature hepatocyte, the phenotypes of CK19+/GPC3+, CK19−/GPC3+, and CK19−/GPC3− can roughly correspond to the HPC, immature hepatocyte, and terminally differentiated hepatocyte, respectively^24^. The hierarchal HCC subtypes in turn might have been transformed from the varied differentiation stages of hepatic parenchymal cells and can fall into the above three discussed immune-phenotype groups.

Previously, a more precise sub-classification of HCC was defined by combined detection of CK19 and GPC3, which produced a more efficient stratification for the microvascular involvement, regional lymph node, intrahepatic and extrahepatic metastasis of patients^25^. Later, another study reported that CK19/GPC3 sub-typing had good predictive performance for post-LT recurrence of HCC patients who fulfilled the MC^26^. Patients with a single nodule greater than 5 cm were excluded by the MC. However, some of them might benefit from LT. In view of the heterogeneous constitution and distinct outcomes of the patients with solitary HCC, a more precise evaluation of disease is needed. Hence, in the present study, a model combining CK19 and GPC3 as a predicting system for early recurrence of solitary HCC was developed. The performance of the model was tested using a validation cohort. Also, the predictive capability of the model was compared with that of several major HCC staging systems that are in use today in predicting the probability of HCC recurrence after LT.

## Materials and Methods

### Subjects

Organ donation or transplantation in the study was strictly implemented under the regulation of the China Organ Donation Committee (CODC), Organ Transplant Committee (OTC) and the Declaration of Helsinki (1983). All recipients received graft from deceased donors. These organs were obtained from 86 academic hospitals or transplant centers in mainland China and registered in a legal organ donation system, known as the China Liver Transplant Registry (CLTR), which was set up in 2005 and regulated by the Chinese government. No organs were procured from (executed) prisoners. All patients provided written informed consent for the collection of samples, information and subsequent analysis. The study protocol was approved by the Ethics Committee of Beijing You-An Hospital, Capital Medical University[No. 2005-01-10].

**Figure 1:**
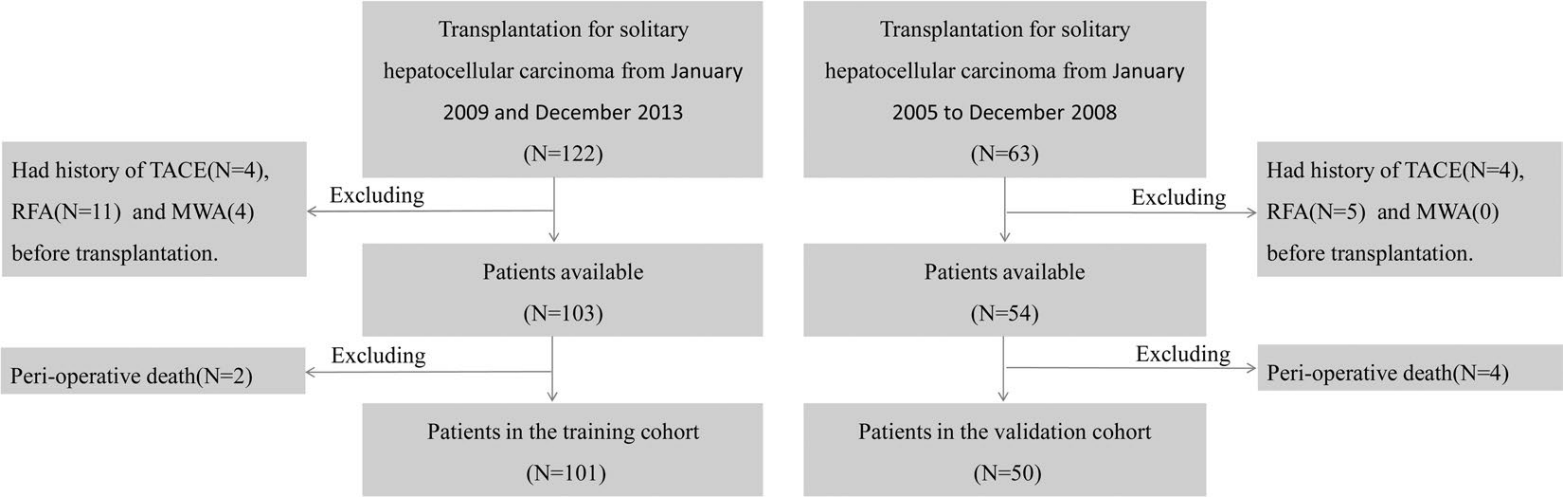
Flow chart of patient selection procedure. TACE, transarterial chemoembolization; RFA, radiofrequency ablation; MWA, microwave albation.

Between January 2004 and December 2013, a total of 185 solitary HCC patients who underwent OLT, with deceased donor at You-An Hospital, Capital Medical University were consecutively enrolled in this retrospective study. The medical imaging data and pathological data of patients were obtained from the medical imaging center and pathology center of the hospital, respectively. Solitary HCC cases were confirmed by preoperative dynamic contrast enhanced magnetic resonance imaging (DCE-MRI) scan without any evidence of extrahepatic or nodal disease. Tumor size was determined by the longest diameter in the DCE-MRI. All explanted liver were pathologically confirmed to have no multiple nodules in this study. Vascular invasion include macroscopic vascular invasion and microscopic vascular invasion. In this study, macroscopic vascular invasion was determined by pathological examination in the explanted liver despite the absence in preoperative images. Microscopic vascular invasion was defined by the presence of tumor emboli within the central hepatic vein, the portal, or the large capsular vessel under the microscope. Cirrhosis was determined based on pathological findings. Throughout the study period, patients were included in the waiting list for LT if meeting the MC or BCLC stage A (1–4) in our center. Solitary HCC patients with cirrhosis and portal hypertension are eligible for liver transplantation directly. These patients can simultaneously fall within the UCSF or other two well known standards for LT in China, Fudan and Hangzhou criteria^27^. All the 185 patients were newly diagnosed with HCC. According to the selection criteria (Fig. 1), 34 patients were excluded because of the preoperative transarterial chemoembolization, ablation therapy or perioperative death (within one month of LT). Based on the operation time, 101 patients who accepted LT between July 2007 and December 2013 were assigned to the training cohort, and 50 patients between January 2004 and June 2007 were assigned to the validation cohort.

### Surgical procedures

All procedures were performed by the same group of experienced surgeons who were trained in the same dissection and reconstructive methods^28^. OLT was performed using an ABO-compatible liver graft from brain-dead standard criteria donor, and the same technique was followed in all the patients. Triple regimen immunosuppression was followed, which consisted of tacrolimus capsules(FK506,Astellas Ireland Co.Ltd, Dublin, Ireland), mycophenolate mofetil (MMF, Shanghai Roche Pharmaceuticals Ltd, Shanghai, China) and methylprednisone (Pfizer Manufacturing Belgium NV, Puurs, Belgium)^29^. FK506, with an initial dose of 0.1 mg/(kg.d) twice daily, was orally administered at 24th hours after operation. The daily dose was then adjusted to reach the following target levels: 8 to 12 ng/mL during the first month, 7 to 10 ng/mL during the first year, and 3 to 7 ng/mL thereafter according to the patient’s graft tolerance. Methylprednisolone (10 mg/kg) was intravenously infused during operation. After surgery, it was given intravenously by 200 mg/day, and reduced by 20% daily till 6th day. Steroids were then given orally, 20 mg per day, and weekly reduced by 25% till the 4th week (5 mg/day), and maintained this dose for two months. Patients were withdrawn from steroids after 3 months post-LT. MMF was administrated orally by 1000 mg/day after operation, and withdrawn 3 months later. Patients with rejection episodes were treated with high-dose methylprednisone (1000 mg methylprednisolone was intravenously infused on the 1st day, 500 mg on the 2nd day, then the dose was tapered by 40 mg every day for 5 days) and short courses of basiliximab (Simulect; Novartis Pharma Inc, Basel, Switzerland) were intravenously infused with two doses of 20 mg over 20 minutes with an interval of 4 days. After LT, no other anticancer treatment was administered to the patients until recurrence.

### HBV or HCV prophylaxis

For patients with HBV infection, 8000 IU HBIG was intravenously given at the time of transplant during the anhepatic phase. Recipients received 400 IU one time only for HBsAb titer <200 IU/L. After six months, anti-hepatitis B immunoglobulin was administered when HBsAb titers fell below 100 IU/L. One hundred milligrams of lamuvidine or 10 milligrams entecavir was taken orally a few hours post-transplant and recipients were maintained on this dose daily. In our cohorts, patients with preoperative positive serum HCV RNA results were treated with interferon. Serum HCV RNA was detected postoperatively, and if HCV RNA titer ≥10^3^ copy/mL, antiviral therapy was started one month after the surgery.

### Immunohistochemical (IHC) staining

Hematoxylin and eosin-stained slides and formalin fixed paraffin embedded blocks of HCC were retrieved from the archives of the Department of Pathology, Beijing You-An Hospital, Capital Medical University. IHC staining was performed as described previously^30^. Mouse anti-human CK19 monoclonal antibody (Clone BA17; Dilution, 1:100) and mouse anti-human GPC3 monoclonal antibody (Clone 1G12; Dilution, 1:200) were purchased from Zeta Company (Sierra Madre, CA, USA). The samples were then steamed for 20 minutes in citrate target retrieval buffer (pH 6.0). The results of IHC were interpreted as positive if greater than 5% of the tumor cells as shown by cytoplasmic staining for CK19 or GPC3^16^. All the enrolled cases were divided into three groups: CK19+/GPC3+ group, which included tumor cells that co-expressed CK19 and GPC3; CK19−/GPC3+ group, which included tumor cells that expressed GPC3 alone; and CK19−/GPC3− group, which included tumor cells without expression of both CK19 GPC3^25^. Positive immunoreactivity of normal bile duct epithelium was confirmed as internal positive control for CK19, while yolk sac tumor tissue was used as a positive control sample for GPC3^24^. Negative controls were carried out by substitution of the primary antibodies with non-immunized serum, which resulted in no signal detection. All slides were assessed independently and blindly by two pathologists. A consensus was reached by careful discussion in case of any disagreement between the two evaluators. The diagnosis and histological grading of HCC was made based on the guidelines of World Health Organization (WHO) criteria, 2010^31^.

### Serum alpha-fetoprotein (AFP) measurement

All AFP values analyzed in the current study were obtained within 24 hours prior to transplantation. Serum AFP concentrations were analyzed using the Elecsys 2010 System (Roche Diagnostics, Mannheim, Germany) according to the manufacturer’s instructions.

### Follow-up

All patients were followed up at our outpatient clinic or through a mobile social media interview. The survivors were regularly followed up at the clinic: monthly during the first 6 months after OLT, every 3 months from seventh to 18th month, and every 6 months thereafter. Abdominal/pelvic ultrasonography and chest X-ray were routinely performed during follow-up. When suspected metastasis or recurrence, further evaluations were made by DCE-MRI or contrast enhanced computed tomography scan and, if necessary, biopsy was performed to confirm the diagnosis^32^. The outcome of this study was the recurrence of HCC after OLT within 3 years. Recurrence-free survival (RFS) time was calculated from the date of OLT to the date of a suspected tumor recurrence in patients with eventually confirmed tumor recurrence or till the last follow-up contact in patients without tumor recurrence. Patients who died before experiencing the disease recurrence or lost to follow up were considered censored. The cut-off date for the follow-up visit was 1st July 2017.

### Statistical analysis

All analyses were performed using SPSS (version 21.0, SPSS, Chicago, IL). Continuous data were presented as mean ± SD or median (range), whereas categorical data by frequency and percentage. χ^2^ test and Student t test were applied to compare the distribution of categorical and continuous variables, respectively. Survival curves were constructed using the Kaplan-Meier method and the differences between the curves were assessed using log rank test. The best cutoff values for serum AFP level and tumor diameter for recurrence was defined as the point with maximum Youden’s index (sensitivity + specificity − 1) on the ROC curve. Univariable and multivariable Cox proportional hazards regression analysis was used to assess the factors associated with recurrence. The significant factors (P < 0.05) in the univariate analysis were assessed for multicollinearity before performing multivariate analyses. A tolerance of less than 0.20 and/or a variance inflation factor (VIF) of 10 and above indicates a multicollinearity problem^33^. The variables without multicollinearity problem were included in the multivariate analysis, and was conducted using the Cox proportional hazards regression model using a backward stepwise approach to identify independent predictors of recurrence. A scoring model was devised on the basis of Cox regression results. The reference for each variable was assigned a value of zero. For the remaining values of the variables, the lowest beta coefficient was given a value of 1, and the other coefficients were adjusted proportionally and rounded to the nearest integer^34^. The summary risk score for an individual was obtained by summing the weighted scores of each of the risk factors. The threshold of the risk index for recurrence prediction was determined by the maximum value of Youden’s index in the ROC curve. Overall predictive performance was measured by AUC of the ROC curve, with 0.5 and 1.0 indicating no and perfect predictive ability, respectively. Hanley-McNeil method was used to test the statistical significance of the difference between the AUCs. All P values were two tailed, and P < 0.05 was considered to be statistically significant.

## Results

### Baseline characteristics of patients in the training cohort

The clinical and tumor characteristics of patients in the training cohort are listed in Table 1. Briefly, the cohort consisted of 11 CK19+/GPC3+ cases, 56 CK19−/GPC3+ cases, and 34 CK19−/GPC3− cases, accounting for 10.9%, 55.4%, and 33.7% of the training data set, respectively. The macroscopic and microscopic vascular invasion occurred in 17 patients (16.8%) and 52 patients (51.5%), respectively. Within three years after LT, total recurrence rate of patients was 29.7% (30/101) in this cohort. The median time to recurrence was 10.5 months (range, 2–24). The median follow-up period was 24 months (range: 1–120 months). Two cases of perioperative death, including one from serious infection and one from renal failure were excluded.

**Table 1:** Baseline clinical characteristics of patients in the training cohort.

Variable		Recurrence	No
Age, years	Median (range)	59 (52–68)	49 (29–69)
	mean ± SD	59.7 ± 4.5	50.7 ± 9.4
Gender, n (%)	male	26 (25.7)	57 (56.4)
	female	4 (4.0)	14 (13.9)
Cirrhosis, n (%)	yes	27 (26.7)	69 (68.3)
	no	3 (3.0)	2 (2.0)
Etiology, n (%)	HBV infection	29 (28.7)	60 (59.4)
	HCV infection	1 (1.0)	5 (5.0)
	alcohol abuse	0 (0)	2 (2.0)
	Budd-Chiari syndrome	0 (0)	1 (1.0)
	schistosome infection	0 (0)	1 (1.0)
	HBV infection + alcohol abuse	0 (0)	1 (1.0)
	HBV + HEV infection	0 (0)	1 (1.0)
	HBV + HCV infection	0 (0)	0 (0)
	AIH	0 (0)	0 (0)
Child-Pugh score	A	20 (19.8)	41 (40.6)
	B	8 (8.0)	26 (25.8)
	C	2 (2.0)	4 (4.0)
MELD score	Median (range)	7 (3–30)	8 (1–25)
AFP level	≤20 ng/mL	5 (5.0)	34 (33.7)
	>20 ng/ mL, ≤200 ng/mL	3 (3.0)	16 (15.8)
	>200 ng/ mL, ≤400 ng/mL	3 (3.0)	8 (7.9)
	>400 ng/ mL	19 (18.8)	13 (12.9)
CK19/GPC3 sub-typing, n (%)	CK19+/GPC3+	8 (7.9)	3 (3.0)
	CK19−/GPC3+	21 (20.8)	35 (34.7)
	CK19−/GPC3−	1 (1.0)	33 (32.7)
Histological grading, n (%)	poorly	16 (15.8)	19 (18.8)
	moderately	13 (12.9)	35 (34.7)
	well	1 (1.0)	17 (16.8)
Macroscopic vascular invasion, n (%)	yes	11 (10.9)	6 (5.9)
	no	19 (18.8)	65 (64.4)
Microscopic vascular invasion, n (%)	yes	23 (22.8)	29 (28.7)
	no	7 (6.9)	42 (41.6)
Tumor diameter, n (%)	≤3 cm	9 (8.9)	54 (53.5)
	>3 cm, ≤5 cm	6 (5.9)	11 (10.9)
	>5 cm	15 (14.9)	6 (5.9)
Milan criteria, n (%)	within	15 (14.9)	65 (64.4)
	beyond	15 (14.9)	6 (5.9)
UCSF criteria, n (%)	within	20 (19.8)	68 (67.3)
	beyond	10 (9.9)	3 (3.0)
Fudan criteria, n (%)	within	24 (23.8)	70 (69.3)
	beyond	6 (5.9)	1 (1.0)
Hangzhou criteria, n (%)	within	25 (24.8)	69 (68.3)
	beyond	5 (5.0)	2 (2.0)

SD, standard deviation; HBV, hepatitis C virus; HCV, hepatitis C virus; CK19, cytokeratin 19.

GPC3, glypican 3; UCSF, University of California, San Francisco.

### ROC curve of AFP level, tumor diameter and model for end-stage liver disease (MELD) score in recurrence prediction

**Figure 2:**
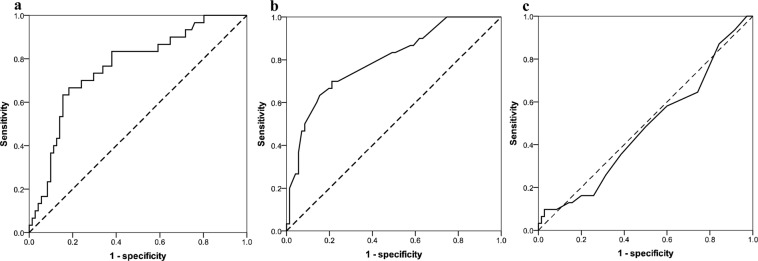
Receiver-operating characteristic curve showed discrimanative performance of alpha-fetoprotein (**a**), tumor diameter (**b**), and MELD score (**c**).

In the training cohort, 39 patients had a serum AFP level of ≤20 ng/mL, accounting for 38.6% of the dataset. The median AFP value was 92.3 ng/mL (range, 1.27–65236). When the cutoff was determined for the point on the ROC curve that maximizes sensitivity (70.0%) and specificity (76.1%) by Youden’s index, the optimal AFP cut-off value for tumor recurrence was 261.6 ng/mL [AUC = 0.730 (95% CI = 0.619–0.842); P < 0.001], (Fig. 2a).

Eighty patients had a tumor diameter of ≤5 cm, which accounted for 79.2% of all patients in this dataset. The median tumor diameter was 3.0 cm (range, 0.5–22). ROC analysis showed that the optimal cutoff value for tumor recurrence was 3.6 cm [AUC = 0.744 (95% CI = 0.634–0.855); P < 0.001] with a sensitivity of 70.0%, and specificity of 78.9% (Fig. 2b).

The MELD based evaluation system was commonly implemented worldwide for predicting the survival after LT. In the training cohort, the median MELD value was 7 (range, 1–30). The ROC curve analysis showed the poor discriminative ability of the MELD score for recurrence prediction [AUC = 0.477, (95% CI = 0.355–0.600); P = 0.718] (Fig. 2c).

### Univariate analyses of RFS in HCC patients

**Figure 3:**
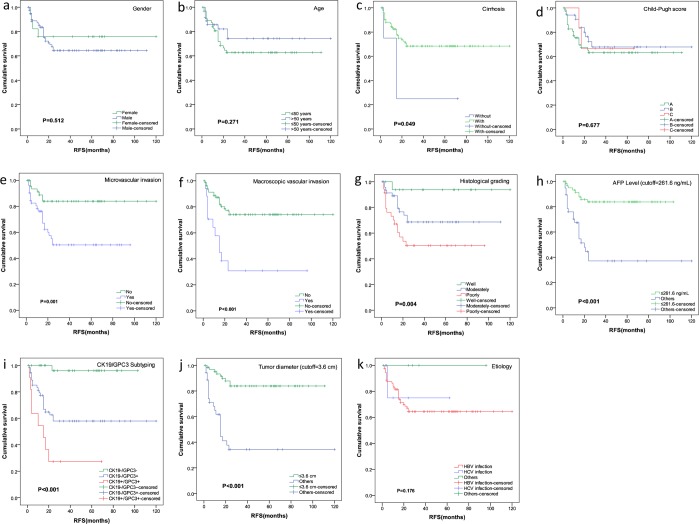
Univariate analyses of RFS in the training cohort.

The RFS was compared for eleven possible prognostic factors, including gender, age, etiology, presence of cirrhosis, Child-Pugh score, histological grading, CK19/GPC3 sub-typing, tumor diameter, AFP level, macroscopic and microscopic vascular invasion. Using the Kaplan–Meier method, univariate analysis showed that seven factors, including cirrhosis, macroscopic and microscopic vascular invasion, histological grading, CK19/GPC3 sub-typing, AFP level >261.6 ng/mL and tumor diameter >3.6 cm were significantly associated with RFS of patients in the training cohort (Fig. 3).

### Multivariate Cox regression analyses

The predictor variables were assessed for multicollinearity before performing multivariate analyses. The VIF was below the limit of 10 and the tolerance was above the crucial threshold of 0.2 for all groups, which indicated that there was no problematic multicolinearity among the main factors (Supplemental Table 1). Therefore, seven variables in the training cohort that were significant in the univariate analysis entered multivariate analysis. As shown in Table 2, the CK19/GPC3 sub-typing [CK19−/GPC3+: hazard ratio (HR) = 6.413, 95% CI = 0.775–48.693, *P* = 0.086; CK19+/GPC3+: HR = 49.054, 95% CI = 5.533–434.882, *P* < 0.001], microscopic vascular invasion (HR = 1.210, 95% CI = 1.393–8.072, *P* = 0.007), AFP level >261.6 ng/mL (HR = 2.434, 95% CI = 1.060–5.594, *P* = 0.036) and tumor diameter >3.6 cm (HR = 3.221, 95% CI = 1.973–5.259, *P* < 0.001) were shown to be independent predictors of RFS.

**Table 2:** Multivariate Cox regression analysis and integer score assignment algorithm based on the β-coefficients.

Variable	β-coefficient	HR	95% CI (Lower-Upper)	*P* value	Score
CK19/GPC3 sub-typing					
CK19−/GPC3−		Reference		<0.001	0
CK19−/GPC3+	1.815	6.143	0.775–48.693	=0.086	2
CK19+/GPC3+	3.893	49.054	5.533–434.882	<0.001	4
Microscopic vascular invasion					
No		Reference		=0.007	0
Yes	1.210	3.353	1.393–8.072		1
Tumor diameter					
≤3.6 cm		Reference		<0.001	0
>3.6 cm	1.170	3.221	1.973–5.259		1
AFP level					
≤261.6 ng/mL		Reference		=0.036	0
>261.6 ng/mL	0.890	2.435	1.060–5.594		1

HR: hazard ratio; CI: confidence intervals; CK19: cytokeratin 19; GPC3: glypican 3.

### Risk scoring for predicting the recurrence of HCC patients after LT

**Figure 4:**
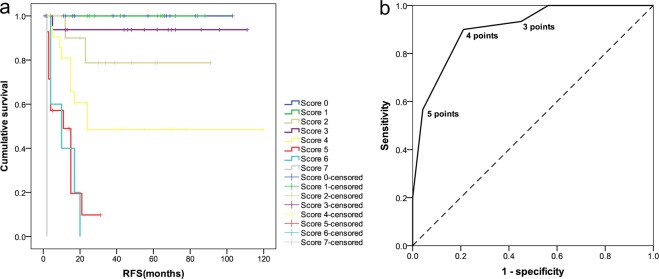
Developmemt of a recurrence prediction model. According to the survival curve, the observation groups were naturally categorized into two risk classes: low risk (0–4 points) and high risk (5–7 points) (**a**); High and low risk patients are easily identified at either end of the ROC curve (**b**).

**Figure 5:**
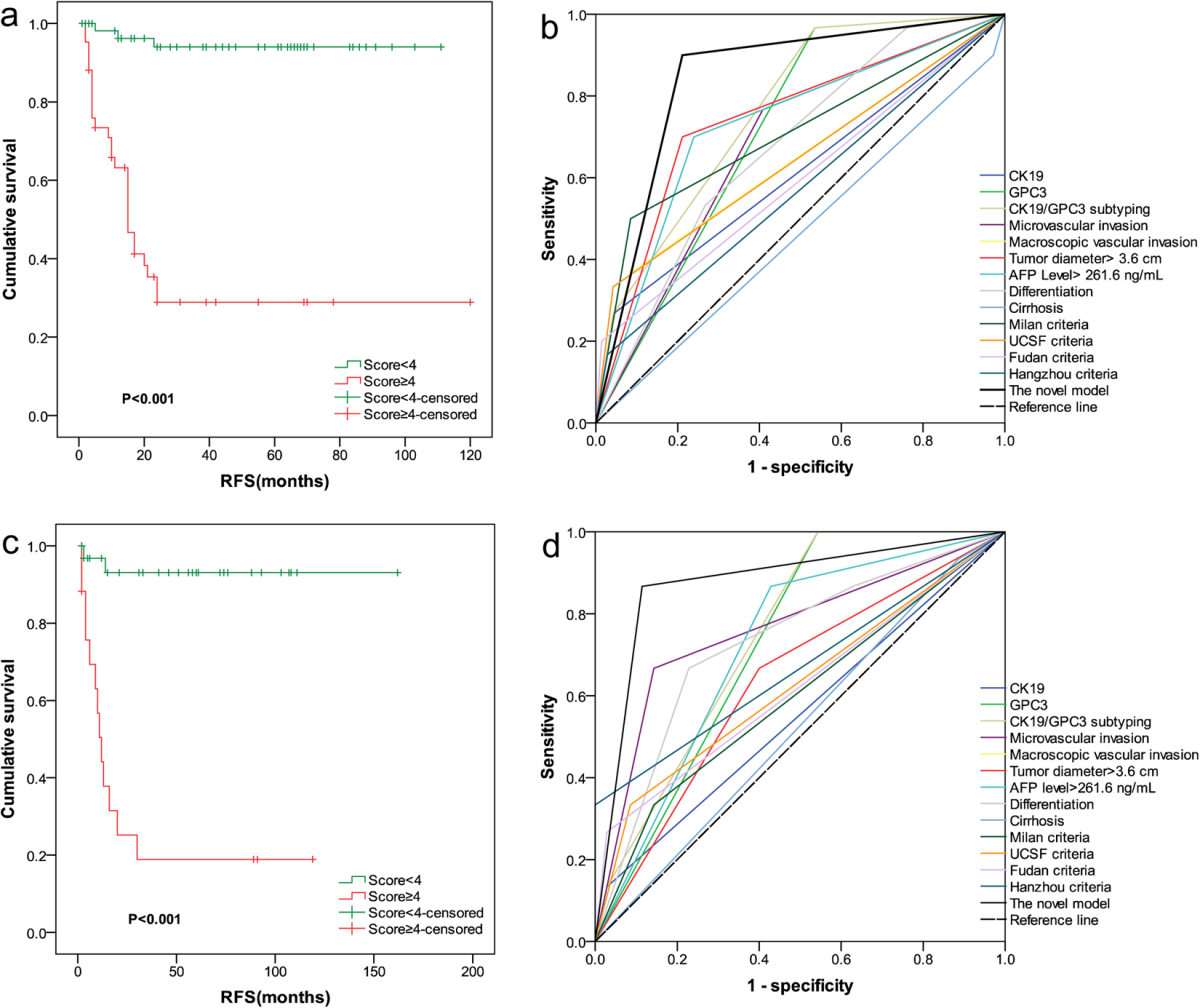
Recurrence-risk stratification and predictive performance of the model in the training and validation cohorts. The log-rank test showed a significant difference among the survival curves of the two groups in the training cohort (**a**) and validation cohort (**c**). The AUC values of recurrence prediction model were highest in the training cohort (**b**) and validation cohort (**d**) compared to the other factors and patient selection criteria.

According to the results of multivariate Cox regression analysis, four independent predictors entered the risk scoring model. A score for each predictor was assigned based on its coefficient value: patients with tumor diameter under the cut-off value of 3.6 cm were assigned 0 points and 1 points for the others. HCC with CK19+/GPC3+, CK19−/GPC3+ and CK19−/GPC3− expression was assigned 4, 2 and 0 points, respectively. HCCs with AFP level <261.6 ng/mL were assigned 0 points, and 1 point for the others. Patients with vascular invasion were assigned 1 point and 0 points for the others. The values of these four variables were then added to get a final score. The total possible score points was 7. As shown in Fig. 4, the score points fell naturally into three risk classes according to the RFS rate that was observed within them: low risk (0–4 points) and high risk (5–7 points) (a). High and low risk patients are easily identified at either end of the ROC curve (b). As shown in Fig. 5a, the log-rank test showed a significant difference between the survival curves of any two groups. At 24 and 36 months after LT, the mean recurrence rates at low risk and high risk groups were 5.1% and 64.3%, respectively (Table 3).

**Table 3:** Recurrence-free survival analysis by risk score in the two cohorts.

Groups of risk	Training cohort						Validation cohort					
	Estimated RFS rates				Mean recurrence rates	*P*	Estimated RFS rates				Mean recurrence rates	*P*
	n	12 month	24 month	36 month			n	12 month	24 month	36 month		
Low	59	96.2%	94.0%	94.0%	5.1%	<0.001	33	96.8%	93.1%	93.1%	6.1%	<0.001
High	42	63.2%	28.9%	28.9%	64.3%		17	44.1%	25.8%	18.9%	76.1%	

RFS: recurrence-free survival.

### Comparing the performance of the model with single predictors and other criteria in the training cohort

As shown in Fig. 5b and Supplemental Table 2, the AUC value of the novel model was 0.844 (95% CI = 0.760–0.929; *P* < 0.001), which was highest compared to that of any single predictors and patient selection criteria in the training cohort. To confirm the superiority of the novel model, Hanley-McNeil method was used to test the statistical significance of the difference in AUC between our model and other selection criteria for patients with HCC. The results shows that our model significantly improved the predictive capacity for the post-transplant recurrence of solitary HCC patients who meet UCSF criteria (=0.007), Fudan criteria (=0.005), and Hangzhou criteria (=0.002) in the training cohort. A statistical difference close to significance was found in AUC between our model and Milan criteria (p = 0.052) (Supplemental Table 3).

### Confirmation of prognostic ability in the validation cohort

To test the accuracy of the model prediction, another independent validation cohort consisting of 50 HCC patients after LT was investigated. The clinical and tumor characteristics of patients in the validation cohort are listed in Supplemental Table 4. The training and validation groups were similar in baseline characteristics, indicating that there was no potential sample selection bias between them that might be caused by the surgeon team’s surgical experience and skills at different times. Briefly, the cohort consisted of 3 CK19+/GPC3+ cases, 31 CK19−/GPC3+ cases, and 16 CK19−/GPC3− cases, accounting for 6.0%, 62.0%, and 32.0% of the dataset, respectively. The microscopic and macroscopic vascular invasion was found in 15 patients (30.0%) and 1 patient (2%), respectively. Within three years after LT, total recurrence rate of patients was 30.0% (15/50) in this cohort. Median time to recurrence was 10 months (range, 2–30). The median follow-up period was 32 months (range: 2–162) in the validation cohort. Four cases of perioperative death, including 2 from severe infection, 1 from biliary ischemia, and 1 from pulmonary emboli were excluded.

As shown in Fig. 5c, similar prognostic results of the model in this cohort were observed. At 36 months after LT, the mean recurrence rates at low- and high-risk groups were 6.1% and 76.1%, respectively. Kaplan–Meier analysis showed significant difference in RFS rates between low and high risk groups: P < 0.001) (Table 3). Furthermore, performance of the model was compared to that of other predictors and patients selection criteria in the validation cohort. As expected, the novel model demonstrated a high degree of discrimination in this cohort with an AUC value of 0.876 (95% CI:0.759–0.994, p < 0.001), which was highest compared to that of the other predictors or patients selection criteria (Fig. 5d, Supplemental Table 2). Hanley-McNeil test showed that the AUC of the novel model was significantly higher than that of the Milan criteria, (p = 0.005), UCSF criteria (p = 0.012), and Fudan criteria (p = 0.014), and Hangzhou criteria (0.045) (Supplemental Table 3).

## Discussion

Patient with a solitary tumor means a relatively low probability of extrahepatic diffusion from the primary lesion. The present study proposed a novel model to stratify the risk of early recurrence in patients with solitary HCC after LT. By introducing molecular indicator, CK19/GPC3 and AFP, the model showed significantly improved recurrence stratification of these patients who met the major HCC staging systems, including MC, UCSF, Fudan and Hangzhou criteria.

The prognostic significances of CK19 or GPC3 in patients with HCC have been emphasized previously^17–20^. In this work, regression analyses and AUC of CK19/GPC3 sub-typing showed that combined detection of the two markers produced an improved discriminatory power than used alone, which was consistent with our previous studies where we showed that the cellular origin of differentiation was closely linked to the aggressive biological behavior of tumor cells in HCC^18^. Hence, the mechanism of influence of CK19/GPC3 sub-typing towards the aggressive biological behavior of tumor cells should be focused. In HCC, the somatic mutations of *gpc3* or *ck19* genes or their modulators were rarely reported, though it is well known that accumulated mutations are prerequisite for malignant transformation. All the evidence implied that distinct aggressive biology among CK19/GPC3 subtypes in HCC can be determined by epigenetic mechanisms. And a more aggressiveness biology of CK19+/GPC3+ HCC can be inherited from its normal counterpart, HPC, which possess a strong ability to migrate and home^35^.

The importance of AFP level in predicting recurrence of HCC after LT has been emphasized in many models. In our model, the optimal cutoff value of AFP level for recurrence prediction was 261.6 ng/mL, which was lower than that of the Hangzhou criteria (400 ng/mL)^36^, as well as other recurrence predicting models (800–1000 ng/mL)^37–39^. In HCC, the AFP level depends not only on the status of the cellular origin but also on the tumor burden. So, in modeling, the significance of this indicator was determined by its relative weight to other risk factors. In our model, AFP level was used as a risk indicator, and not as an exclusion indicator. As a result, for those patients with AFP > 261.6 ng/mL and no recurrence during follow-up, our classifier was able to identify them satisfactorily.

In our model the optimal cutoff value of tumor diameter for recurrence was 3.6 cm, which was even less than that of the MC. Similar to AFP, this indicator also acts as a risk indicator, not an exclusion indicator. According to our classifier, there were 8 cases with tumor diameter greater than 5 cm (training cohort: 7.9 cm, 8.0 cm, 9.0 cm; validation cohort: 6.0 cm, 6.0 cm, 7.0 cm, 8.0 cm, 14.0 cm) that fell in the low recurrence risk group without recurrence during the follow-up. In addition, in patients with tumors smaller than 5 cm in diameter, 21 cases fell in the high-risk groups (median: 3.7 cm; range, 1.5–5.0 cm) showed recurrence after LT during the follow-up, respectively. Over the past twenty years, MC was applied as the gold standard indication for LT in patients with HCC worldwide^40^. The results of this study indicated that our model not only extended the MC, but also improved the accuracy of recurrence prediction of patients with solitary HCC. By introducing the CK19/GPC3 indicator, we have previous proposed a recurrence prediction model for patients with HCC who meet the MC after LT^26^. That model did not take into account patients beyond the MC, some of whom might have benefited from LT. The novel model proposed in this study partially complements our previous work.

Currently, needle biopsy was commonly used as a safe, rapid and reliable method for HCC diagnosis^41^. Imaging technology and immunohistochemistry together can evaluate tumor number, size, as well as the CK19/GPC3 expression preoperatively. Although vascular invasion is a histopathologic diagnosis and cannot be made prior to the removal of the liver specimen, previous reports showed that an approximately 25–30% of patients could be found with microvascular invasion on preoperative biopsy^42^. Once microvascular invasion in preoperative needle biopsy was found, our model can be used for patient selection for LT. Hence, more evidence is needed to clearly support the hypothesis in future.

The main limitations of this work include its retrospective nature and the patient selection bias caused by the study design. Although competing risk model was recently suggested to be a more reasonable analytical method for dealing with multiple potential outcomes, given the small sample size and the small number of non-HCC deaths, we used standard Cox model. In addition, the efficiency of the novel model should be validated by external cohort. Finally, the cohorts of this study consisted of HBV dominant recipients who underwent deceased donor LT. Predictability may be different in recipients with various etiologies and in recipient undergoing living donor LT.

## Conclusion

Combined CK19/GPC3 sub-typing, pre-transplant AFP level, tumor diameter and vascular invasion for novel risk score model were devised. The model could be used to predict the early recurrence of patients with solitary HCC after LT.

## Supplementary information

Supplementary materials
