# Supplementary material for: Prediction of Early Recurrence of Solitary Hepatocellular Carcinoma after Orthotopic Liver Transplantation
Source: Sci Rep. 2019 Nov 1;9:15855. doi: 10.1038/s41598-019-52427-8 (PMC6825189; doi:10.1038/s41598-019-52427-8)
Supplement: Supplementary file 1 — Supplementary materials [file 41598_2019_52427_MOESM1_ESM.pdf]

Supplementary materials

Title: Prediction of Early Recurrence of Solitary Hepatocellular Carcinoma after Orthotopic Liver Transplantation

Authors: Jiliang Feng[1†], Ruidong Zhu[2†], Dezhao Feng[3], Lu Yu[1], Dawei Zhao[4], Jushan Wu[2], Chunwang Yuan[5], Junmei Chen[6], Yan Zhang[1], Xiu Zheng[1]

Table of contents：

Supplemental Table 1. Multicollinearity analysis

Supplemental Table 2. Comparing the performance of the model with single predictors and other criteria in the two cohorts

Supplemental Table 3. Test the statistical significance of the difference between the AUCs

Supplemental Table 4. Baseline clinical characteristics of patients in the validation cohort

Supplemental Table 1: Multicollinearity analysis

Variable	Adjusted R^2	Unstandardized Coefficients B	Unstandardized Coefficients *Std.* Error	Standardized Coefficients Beta	t	*P*	Tolerance	VIF
**Cirrhosis**	0.404	-0.116	0.175	-0.055	-0.667	0.507	0.868	1.153
**Macroscopic vascular invasion**		0.095	0.111	0.077	0.851	0.397	0.718	1.392
**Microscopic vascular invasion**		0.142	0.079	0.156	1.793	0.076	0.789	1.268
**Histological grading**		0.029	0.058	0.045	0.499	0.619	0.738	1.355
**CK19/GPC3 sub-typing**		0.217	0.067	0.298	3.216	0.002	0.695	1.439
**AFP level**		0.152	0.085	0.162	1.803	0.075	0.742	1.348

**Tumor diameter**	0.274	0.088	0.287	3.107	0.003	0.698	1.433

Dependent Variable: recurrence; VIF, variance inflation factor; *Std.* Error, Standard error; CK, cytokeratin; GPC3, glypican 3; AFP, alpha fetoprotein.

A tolerance of less than 0.20 and/or a VIF of 10 and above indicates a multicollinearity problem.

Supplemental Table 2. Comparing the performance of the model with single predictors and other criteria in the two cohorts

Variable	Training cohort				Validation cohort			
	AUC	95% CI(Lower-Upper)		*P*	AUC	95% CI(Lower-Upper)		*P*
CK19 expression	0.612	0.484	0.741	=0.076	0.552	0.371	0.734	=0.560
GPC3 expression	0.716	0.617	0.815	=0.001	0.729	0.592	0.865	=0.011
CK19/GPC3 sub-typing	0.767	0.673	0.861	<0.001	0.750	0.618	0.883	=0.005
Macroscopic vascular invasion	0.641	0.515	0.768	=0.026	0.486	0.300	0.660	=0.874
Microscopic vascular invasion	0.679	0.567	0.791	=0.005	0.762	0.604	0.920	=0.004
Tumor diameter	0.744	0.634	0.855	<0.001	0.633	0.465	0.802	=0.138
AFP level	0.730	0.619	0.842	<0.001	0.719	0.571	0.867	=0.015
Histological grading	0.677	0.567	0.786	=0.005	0.730	0.572	0.887	=0.011
Cirrhosis	0.464	0.338	0.591	=0.570	0.524	0.350	0.697	=0.791
Milan criteria	0.708	0.586	0.829	=0.001	0.595	0.415	0.775	=0.290
UCSF criteria	0.646	0.518	0.773	=0.021	0.624	0.443	0.805	=0.169
Fudan criteria	0.593	0.464	0.722	=0.141	0.619	0.436	0.802	=0.186
Hanzhou criteria	0.569	0.441	0.697	=0.273	0.667	0.485	0.848	=0.064
The novel model	0.844	0.760	0.929	<0.001	0.876	0.759	0.994	<0.001

AUC, area under the curve; CI, confidence interval; CK, cytokeratin; GPC3, glypican 3; AFP, alpha-fetal protein; UCSF, the University of California

Supplemental Table 3. Test the statistical significance of the difference between the AUCs

	The novel model					
	Training cohort			Validation cohort		
**Variable**	**95% CI (Lower-Upper)**		***P***	**95% CI (Lower-Upper)**		***P***
**Milan criteria**	-0.001	0.274	=0.052	0.085	0.476	=0.005
**UCSF criteria**	0.055	0.342	=0.007	0.055	0.450	=0.012
**Fudan criteria**	0.111	0.392	=0.005	0.053	0.461	=0.014
**Hanzhou criteria**	0.131	0.420	=0.002	0.005	0.414	=0.045

Supplemental Table 4. Baseline clinical characteristics of patients in the validation cohort

**Variable**		**Recurrence**	**No**
**Age, years**	Median(range)	53(43-61)	48(35-73)
	mean ±SD	52 ±4.8	50.4 ±8.3
**Gender, n** (%)			
	male	15(30.0)	28(56.0)
	female	0(0.0)	7(14.0)
**Cirrhosis, n (%)**			
	yes	14(28.0)	31(62.0)
	no	1(2.0)	4(8.0)
**Etiology, n (%)**			
	HBV infection	14(28.0)	28(56.0)
	HCV infection	0(0)	4(8.0)

	alcohol abuse	0(0)	0(0)
	Budd-Chiari syndrome	0(0)	0(0)
	schistosome infection	0(0)	0(0)
	HBV infection+ alcohol abuse	0(0)	0(0)
	HBV+HEV infection	0(0)	0(0)
	HBV+HCV infection	1(0)	2(4.0)
	AIH	0(0)	1(2.0)
**Child-Pugh score**	A	8(16.0)	11(22.0)
	B	4(8.0)	18(36.0)
	C	3(6.0)	6(12.0)
**MELD score**	Median(range)	8(2-19)	8(3-27)
**AFP level**			
	≤20 ng/mL	1(2.0)	13(26.0)

	>20 ng/ mL, ≤200 ng/mL	1(2.0)	7(14.0)
	>200 ng/ mL, ≤400 ng/mL	4(8.0)	5(10.0)
	>400 ng/ mL	9(18.0)	10(20.0)
**CK19/GPC3 sub-typing, n (%)**			
	CK19+/GPC3+	2(4.0)	1(2.0)
	CK19-/GPC3+	13(26.0)	18(36.0)
	CK19-/GPC3-	0(0)	16(32.0)
**Histological grading, n (%)**			
	poorly	10(20.0)	8(16.0)
	moderately	3(6.0)	14(28.0)
	well	2(4.0)	13(26.0)

**Macroscopic vascular invasion, n (%)**	yes	0(0)	1(2.0)
	no	15(30.0)	34(68.0)
**Microscopic vascular invasion, n (%)**	yes	10(20.0)	5(10.0)
	no	5(10.0)	30(60.0)
**Tumor diameter, n (%)**			
	≤3cm	5(10.0)	19(38.0)
	>3cm, ≤5cm	5(10.0)	11(22.0)
	>5cm	5(10.0)	5(10.0)
**Milan criteria, n (%)**			
	within	10(20.0)	30(60.0)

	beyond	5(10.0)	5(10.0)
**UCSF criteria, n (%)**			
	within	10(20.0）	32（64.0）
	beyond	5(10.0)	3(6.0)
**Fudan criteria, n (%)**			
	within	11(22.0)	34(68.0)
	beyond	4(8.0)	1(2.0)
**Hangzhou criteria, n (%)**			
	within	10(20.0)	35(70.0)
	beyond	5(10.0)	0(0.0)

SD, standard deviation; HBV, hepatitis C virus; HCV, hepatitis C virus; CK19, cytokeratin 19; GPC3, glypican 3; UCSF, University of California, San Francisco
